# Correlates of Initiation of Treatment for Chronic Hepatitis C Infection in United States Veterans, 2004–2009

**DOI:** 10.1371/journal.pone.0132056

**Published:** 2015-07-13

**Authors:** Adi V. Gundlapalli, Richard E. Nelson, Candace Haroldsen, Marjorie E. Carter, Joanne LaFleur

**Affiliations:** 1 Informatics, Decision Enhancement, and Surveillance (IDEAS) Center, VA Salt Lake City Health Care System, Salt Lake City, Utah, United States of America; 2 University of Utah School of Medicine, Salt Lake City, Utah, United States of America; 3 University of Utah College of Pharmacy, Salt Lake City, Utah, United States of America; University of Montreal Hospital Research Center (CRCHUM), CANADA

## Abstract

We describe the rates and predictors of initiation of treatment for chronic hepatitis C (HCV) infection in a large cohort of HCV positive Veterans seen in U.S. Department of Veterans Affairs (VA) facilities between January 1, 2004 and December 31, 2009. In addition, we identify the relationship between homelessness among these Veterans and treatment initiation. Univariate and multivariable Cox Proportional Hazards regression models with time-varying covariates were used to identify predictors of initiation of treatment with pegylated interferon alpha plus ribavirin. Of the 101,444 HCV treatment-naïve Veterans during the study period, rates of initiation of treatment among homeless and non-homeless Veterans with HCV were low and clinically similar (6.2% vs. 7.4%, p<0.0001). For all U.S. Veterans, being diagnosed with genotype 2 or 3, black or other/unknown race, having Medicare or other insurance increased the risk of treatment. Veterans with age ≥50 years, drug abuse, diabetes, and hemoglobin < 10 g/dL showed lower rates of treatment. Initiation of treatment for HCV in homeless Veterans is low; similar factors predicted initiation of treatment. Additionally, exposure to treatment with medications for diabetes predicted lower rates of treatment. As newer therapies become available for HCV, these results may inform further studies and guide strategies to increase treatment rates in all U.S. Veterans and those who experience homelessness.

## Introduction

Hepatitis C virus (HCV) is a common blood-borne viral infection and a major public health concern that affects nearly 1 to 1.5% of the U.S. population, between 3 and 5 million people [1–3]. Several studies have noted a substantially higher prevalence of HCV (as high as 6.6%) in U.S. military Veterans compared to the general population [4, 5]. Presumably due to the co-occurrence of known risk factors, studies have shown an even higher prevalence among individuals experiencing homelessness in general [2, 6, 7] and homeless Veterans in particular [8–11].

Several studies have assessed the eligibility for and rates of treatment of Veterans with HCV [11–15]. The Department of Veterans Affairs (VA) has published a report on the state of HCV among Veterans [16]. Though screening and treatment rates are slowly increasing and are considered higher than the general U.S. population [17], most Veterans are deemed poor candidates for HCV treatment due to issues with substance abuse, mental health, and medical comorbidities. With the availability of new treatment regimens, being the largest provider of HCV care in the world, the VA “expects to treat all Veterans with chronic hepatitis C virus infection who wish to be treated and are suitable for treatment [18].” Homeless Veterans have not been specifically included in any of these evaluations.

Testing and treatment for hepatitis C has individual and societal benefits. Chronic hepatitis C infection is the leading cause for hepatocellular carcinoma and the leading indication for liver transplantation in the U.S. [3]. Direct acting anti-viral medications that have become available in the last few years have greatly changed the landscape of HCV management [19]. Though expensive, these oral regimens have greatly improved response rates with fewer side effects and require less monitoring.

Our goal was to describe the rates and predictors of initiators of treatment among a large cohort of HCV positive U.S. Veterans during a period when treatment consisted of pegylated interferon (Peg-IFN) alpha plus ribavirin (2004–2009). A second objective was to determine whether homelessness among these Veterans influences initiation of HCV therapy.

## Methods

### Design and datasets

This study used a historical cohort study design with data from a national cohort of U.S. Veterans extracted from the VA Informatics and Computing Infrastructure (VINCI) environment [18]. Specific datasets included Corporate Data Warehouse (CDW) data for demographic data, height, weight, pharmacy records, laboratory test results, and Medical SAS datasets for inpatient and outpatient visits, including procedures and diagnosis codes.

### Cohort Selection

We used data from national VA databases to identify all U.S. Veterans who were seen in VA facilities between January 1, 2004 and December 31, 2009 who also had evidence of HCV infection. This ensured a historical cohort of chronic HCV infected Veterans who had not been exposed to or offered newer treatment regimens. HCV infection was defined using the following ICD-9-CM codes: 070.41 (acute hepatitis C with hepatic coma), 070.44 (chronic hepatitis C with hepatic coma), 070.51 (acute hepatitis C without hepatic coma), 070.54 (chronic hepatitis C without hepatic coma), 070.7* (unspecified viral hepatitis C with and without hepatic coma), and V02.62 (hepatitis C carrier). The date of the first diagnosis was defined as the “index date”. We excluded patients treated with Peg-IFN and/or RBV before the first diagnosis date to ensure treatment naiveté. We also excluded patients who did not have encounters in the VA system at least 180 days prior to the index date to ensure that we identified patients who were regularly seeking care in the VA setting.

To confirm that we identified true chronic HCV infected patients, we excluded patients who did *not* have either (a) a second diagnosis for HCV on a different date within 395 days of the first (to ensure capture of yearly follow-ups with a one month variance), or (b) a confirmatory lab result within 6 months before or 395 days after the date of the first diagnosis. Laboratory values that were considered “confirmatory” included HCV genotype with any valid value (1–7), positive HCV antibody, positive qualitative HCV RNA, or quantitative HCV RNA with any numerical value [20, 21].

### Framework for analysis

The general conceptual and rational framework for this analysis is based on evidence-based work on the natural history of chronic hepatitis C infection and selection of patients for HCV treatment [22]. The criteria for considering treatment such as progression of disease and effects on the liver are as important as the relative and absolute contraindications for excluding patients from therapy [23]. These are conceptualized as those that represent the characteristics of the patient (socio-demographic variables such as age, gender), specific effects of HCV on the liver (extent of liver disease), behavioral factors (alcoholism, drug abuse, tobacco use), interactions with the healthcare system (presence of chronic co-morbid conditions and treatments for those conditions), and medical status (specific laboratory parameters).

A second construct is the observation that individuals and specifically Veterans with evidence of homelessness are noted to have higher rates of HCV, behavioral risk factors, and chronic co- morbid conditions [8–11]. Those who experience homelessness are also at risk for poor general health and adverse outcomes in terms of morbidity and mortality [24, 25].

From administrative databases, these categories are represented as demographic variables (age, gender), co-morbid conditions (ICD-9-CM codes associated with healthcare visits), pharmacy data (prescription for specific medications) and laboratory data (blood test results).

Our objective was to assess the relative risk of the outcome (which is the initiation of treatment) over time across two exposure groups (in this case–homeless vs. housed Veterans). Thus, we favored the use of longitudinal data over cross-sectional data. Furthermore, we performed a time-varying analysis using longitudinal data as the occurrence of medical and laboratory contraindications to therapy are usually noted after confirming the diagnosis of chronic HCV.

### Outcome

The outcome in our analysis was initiation of treatment for chronic hepatitis C infection, defined as a prescription for either PEG-IFN or RBV or both after the index date.

### Independent variables

The key independent variable in our primary analysis was an indicator for homelessness. Homeless Veterans were identified by one or more of the following administrative indicators that have been previously used by the VA [26] and the VA Office of Inspector General [27]: 1) an ICD-9-CM code of V60.0 (lack of housing) recorded as either primary or other code OR (2) any of the following clinic stop codes (which indicates receipt of a specific service for homeless Veterans in VA medical facilities): 522 (Department of Housing and Urban Development-VA Shared Housing (HUD-VASH), 528 (Telephone/Homeless Mentally Ill (HMI)), 530 (Telephone/HUD-VASH), and 590 (Community outreach to homeless Veterans by staff) OR (3) receipt of specific inpatient services such as domiciliary care for homeless Veterans (DCHV, treatment specialty) or Mental Health Residential and Rehab Treatment Program for Compensated Work Therapy/Treatment Resident (MH RRTP CWT/TR, treatment specialty). A Veteran was identified as homeless if there was at least one administrative indicator for homelessness in the 6 months prior to HCV diagnosis date.

Demographic information, co-morbid diagnoses, pharmacy data, and laboratory test results were extracted from existing databases for all Veterans in this cohort during the study period which started 180 days prior to the index date. In this study, co-morbid conditions were assessed using administrative data such as ICD-9-CM codes which are assigned to each patient visit in health care settings and CPT codes which are procedural codes that represent the type of visit and procedure performed at each health care visit. We identified the following contraindications to therapy using ICD-9-CM codes, CPT codes, pharmacy, and laboratory data: uncontrolled seizures, moderate to severe retinopathy, autoimmune hepatitis, bipolar disorder, major depression with suicide attempt, acute coronary syndromes, acute myocardial infarction, liver transplant, kidney transplant, other organ transplant, hepatic decompensation, major hemoglobinopathies, hemoglobin < 10g/dL, neutrophil count < 750/mm^3^, and platelets < 50,000 cells/ mm^3^.

### Analysis

Frequencies of patient characteristics were compared for homeless HCV patients who were treated with Peg-IFN and RBV vs. homeless HCV patients who were not treated during the study period. Univariate and multivariable Cox Proportional Hazards regression models with time-varying covariates were created to identify characteristics that were predictive of treatment with Peg-IFN and RBV; this is represented as a comparison of ‘risk’ of treatment. These regression models were performed for all patients with homelessness as the key independent variable as well as for the subset of those with an indicator for homelessness. We used a combination of forward stepwise and backward selection for the multivariable models, with stepwise elimination if *p*>0.1. Variables were added one at a time, and all previously included variables were retested at each iteration; those with *p*-values <0.1 remained in the model.

As only 11% of our cohort was classified as homeless, the stratified analysis for non-homeless Veterans was nearly identical to the non-stratified analysis that included all Veterans in the cohort. Therefore, to simplify the presentation of the results, we havw only included the overall model and the sub analysis that included only the homeless Veterans.

All statistical analyses were conducted using SAS version 9.2 (SAS Corporation, Cary, NC).

### Ethics Statement

The work described was approved by the University of Utah Institutional Review Board and by the Research and Development committee at VA Salt Lake City Health Care System. A waiver of authorization was approved for retrospective review of existing medical record data. All data were de-identified prior to analysis.

## Results

### Demographics and co-morbid conditions

**Fig 1:**
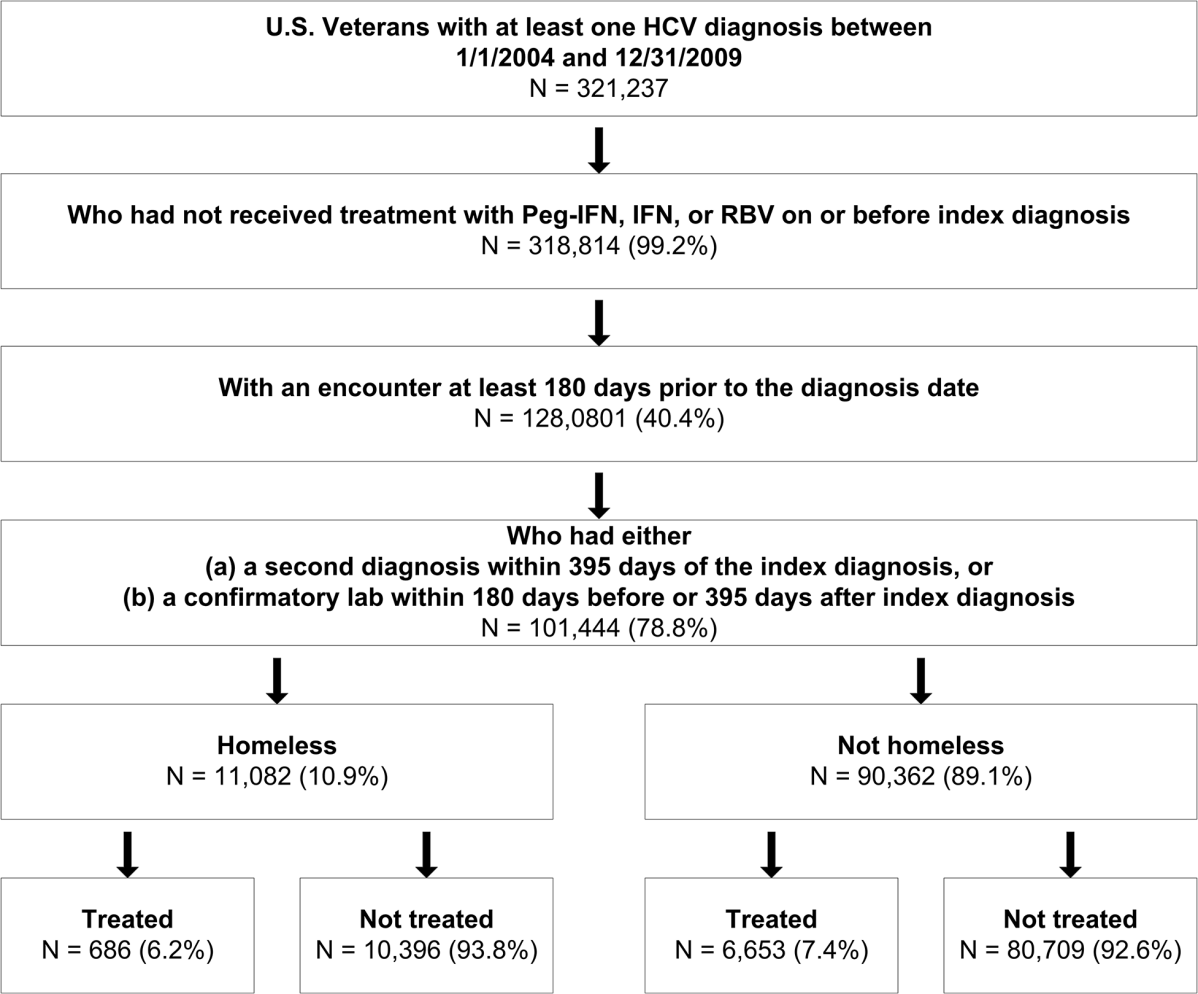
Protocol for identifying HCV positive Veterans who received prescriptions for dual therapy for HCV and their homeless status by administrative indicators, VA medical facilities, 2004–2009.

Of the 321,237 Veterans with a validated diagnosis for HCV in the VA between January 1, 2004 and December 31, 2009, 101,444 (78.8%) were seen in the VA at least 6 months prior to diagnosis date and were treatment naïve (Fig 1).

Table 1 shows the frequency of patient characteristics among Veterans with a diagnosis of HCV who received treatment and those who did not. Overall, treated and untreated patients were significantly different in each of the demographic variables studied. Untreated patients were more likely to have evidence of diagnoses of alcoholism, depression/anxiety/psychosis, drug abuse, and tobacco use.

**Table 1:** Demographic characteristics of 101,444 U.S. Veterans with chronic hepatitis C (HCV) infection who were treatment naïve for HCV, 2004–2009.

	All					Homeless				
	Treated		Not treated			Treated		Not treated		
	N = 8,798		N = 92,637			N = 686		N = 10,396		
										
Variable	N	%	N	%	p-value	N	%	N	%	p-value
**Demographic variables**										
Age										
< 50 (referent)	1,876	21.3%	6,323	6.8%	<0.0001	208	30.3%	1082	10.4%	<0.0001
50+	6,922	78.7%	86,314	93.2%		478	69.7%	9314	89.6%	
Genotype										
1 or 4 (referent)	5,701	64.8%	30,249	32.7%	<0.0001	480	70.0%	3805	36.6%	<0.0001
2 or 3	2,286	26.0%	5,777	6.2%		158	23.0%	600	5.8%	
Other/unknown	811	9.2%	56,620	61.1%		48	7.0%	5991	57.6%	
Marital status										
Married (referent)	5,842	66.4%	66,078	71.3%	<0.0001	609	88.8%	9355	90.0%	0.442
Not married	2,956	33.6%	26,568	28.7%		77	11.2%	1041	10.0%	
Race										
White (referent)	1,757	20.0%	24,288	26.2%	<0.0001	185	27.0%	3450	33.2%	<0.0001
Black	4,516	51.3%	44,658	48.2%		297	43.3%	4761	45.8%	
Other/unknown	2,525	28.7%	23,700	25.6%		204	29.7%	2185	21.0%	
Insurance status										
No insurance (referent)	6,541	74.3%	68,789	74.2%	<0.0001	604	88.0%	9031	86.9%	0.478
Medicare	960	10.9%	12,476	13.5%		41	6.0%	715	6.9%	
Medicaid	95	1.1%	1,132	1.2%		10	1.5%	191	1.8%	
Other	1,198	13.6%	10,149	11.0%		31	4.5%	459	4.4%	
Sex (male)	8,430	95.8%	89,642	96.8%	<0.0001	668	97.4%	10175	97.9%	0.576
**Co-morbid conditions, by ICD-9-CM codes**										
Alcohol	2,125	24.2%	27,603	29.8%	<0.0001	431	62.8%	6,671	64.2%	0.277
Congestive heart failure	72	0.8%	2,626	2.8%	<0.0001	3	0.4%	146	1.4%	0.570
Chronic fatigue syndrome	10	0.1%	92	0.1%	0.629	0	0.0%	3	0.0%	-
Cirrhosis	173	2.0%	3,123	3.4%	<0.0001	11	1.6%	211	2.0%	0.061
COPD	57	0.6%	814	0.9%	<0.0001	2	0.3%	89	0.9%	0.102
Cardiovascular disease	63	0.7%	1,305	1.4%	<0.0001	2	0.3%	92	0.9%	0.079
Dementia	11	0.1%	438	0.5%	<0.0001	1	0.1%	37	0.4%	0.252
Depression/anxiety/psychosis	1,731	19.7%	20,011	21.6%	<0.0001	208	30.3%	3,262	31.4%	0.351
Diabetes	1,283	14.6%	17,565	19.0%	<0.0001	65	9.5%	1,353	13.0%	
Drug abuse	1,924	21.9%	26,643	28.8%	<0.0001	465	67.8%	7,448	71.6%	0.034
HIV	189	2.1%	1,428	1.5%	<0.0001	28	4.1%	299	2.9%	0.046
Liver disease	8,672	98.6%	91,023	98.2%	<0.0001	674	98.3%	10,258	98.7%	0.026
Malig/tumor	268	3.0%	4,142	4.5%	<0.0001	8	1.2%	235	2.3%	
OA	1,272	14.5%	13,088	14.1%	0.742	87	12.7%	1,290	12.4%	0.944
Plegia	66	0.8%	1,023	1.1%	<0.0001	3	0.4%	49	0.5%	0.962
Rheumatoid arthritis	54	0.6%	718	0.8%	0.053	2	0.3%	34	0.3%	0.867
Renal disease	108	1.2%	2,583	2.8%	<0.0001	5	0.7%	150	1.4%	0.065
Sleep apnea	185	2.1%	1,921	2.1%	0.795	4	0.6%	110	1.1%	0.304
Sleep disorders (other)	377	4.3%	4,076	4.4%	0.836	28	4.1%	525	5.1%	0.323
Smoking	2,865	32.6%	31,414	33.9%	0.669	291	42.4%	4,624	44.5%	0.557
Ulcer	100	1.1%	1,262	1.4%	0.011	4	0.6%	116	1.1%	0.102
**Prescriptions for medications to treat the following conditions**										
Alcoholism	39	0.4%	452	0.5%	0.302	8	1.2%	118	1.1%	0.774
Cancer	6	0.1%	81	0.1%	0.476	0	0.0%	2	0.0%	0.667
COPD	481	5.5%	7,670	8.4%	0.000	39	5.7%	839	8.1%	0.013
Dementia	11	0.1%	360	0.4%	0.000	1	0.1%	15	0.1%	0.976
Depression	2,077	23.6%	21,457	23.4%	0.262	223	32.5%	3,463	33.5%	0.245
Diabetes	1,129	12.8%	15,122	16.5%	0.000	49	7.1%	1,194	11.5%	0.000
Drug abuse	58	0.7%	848	0.9%	0.042	21	3.1%	318	3.1%	0.671
HIV	171	1.9%	2,011	2.2%	0.000	9	1.3%	193	1.9%	0.147
Rheumatoid arthritis	0	0.0%	2	0.0%	0.660	0	0.0%	0	0.0%	-
Sleep somnolence	6	0.1%	56	0.1%	0.855	0	0.0%	8	0.1%	0.476
Smoking	855	9.7%	8,558	9.3%	0.009	124	18.1%	1,523	14.7%	0.005
**Contraindications—comorbidities**										
Acute coronary syndrome	32	0.4%	988	1.1%	<0.0001	1	0.1%	85	0.8%	0.050
Autoimmune hepatitis	0	0.0%	12	0.0%	0.380	0	0.0%	0	0.0%	
Bipolar disorder	176	2.0%	3,041	3.3%	<0.0001	27	3.9%	570	5.5%	0.191
Hepatic decompensation	167	1.9%	4,943	5.3%	<0.0001	15	2.2%	443	4.3%	0.010
Intractable epilepsy	5	0.1%	134	0.1%	0.036	1	0.1%	15	0.1%	0.974
Kidney transplant	3	0.0%	160	0.2%	0.002	0	0.0%	7	0.1%	0.476
Liver transplant	42	0.5%	215	0.2%	<0.0001	0	0.0%	5	0.0%	0.544
Major depression with suicide attempt	123	1.4%	1,716	1.9%	0.011	10	1.5%	255	2.5%	0.095
Myocardial infarction	5	0.1%	326	0.4%	<0.0001	1	0.1%	42	0.4%	0.301
Other organ transplant	1	0.0%	4	0.0%	0.442	0	0.0%	0	0.0%	
Retinopathy	91	1.0%	1,957	2.1%	<0.0001	2	0.3%	156	1.5%	0.014
										
**Contraindications—laboratory values**										
Hemoglobin < 10 g/dL	181	2.1%	8,833	9.5%	<0.0001	9	1.3%	795	7.6%	0.014
Neutrophils < 750/mm3	5	0.1%	0	0.0%	<0.0001					
Platelets < 50,000 cells/mm3	40	0.5%	2,478	2.7%	<0.0001	7	1.0%	209	2.0%	0.039

Note: abbreviations: HIV = human immune deficiency virus, OA = osteoarthritis, COPD = chronic obstructive pulmonary disease, additional characteristics that were excluded due to counts ≤ 5 included diagnoses of congestive heart failure, chronic fatigue syndrome, cerebrovascular disease, dementia, plegia, rheumatoid arthritis, renal disease, sleep apnea, and ulcer; exposure to medications used to treat cancer, dementia, insomnia, and rheumatoid arthritis; and the following contraindications to therapy: autoimmune hepatitis, intractable epilepsy, kidney transplant, liver transplant, other organ transplant, myocardial infarction, retinopathy, and platelets < 50,000 cells/mm^3^.

Significant differences between treated and untreated homeless Veterans with a diagnosis of HCV were seen in age, genotype, race, diagnoses of drug abuse, human immune deficiency virus (HIV), malignancy or tumor, exposure to medications used to treat chronic obstructive pulmonary disorder (COPD), diabetes, drug abuse, contraindications such as hepatic decompensation, neutrophil count < 750/mm^3^, and hemoglobin < 10 g/dL. Overall, prevalence of alcoholism (~64%), depression/anxiety/psychosis (~31%), drug abuse (~70%), and tobacco use (~43%) was higher in Veterans with administrative evidence of homelessness as compared to all Veterans. These are representative of individual behavioral risk factors that contribute to accelerated liver disease and also of factors that require more frequent interaction with the health care system for treatment of co-morbid conditions.

### Correlates of initiation of HCV treatment in U.S. Veterans

For all U.S. Veterans, being diagnosed with genotype 2 or 3, black or other/unknown race, having Medicare or other insurance increased the risk of treatment. In addition, those with diagnoses of HIV, liver disease, osteoarthritis, sleep apnea, or exposure to medications to treat insomnia were more likely to receive treatment. Surprisingly, a contraindication of major depression with suicide attempt increased the risk of treatment significantly (HR = 3.567, p<0.0001).

Veterans aged 50 or older, males, and those with other or unknown genotype were less likely to be initiated on treatment. Diagnoses such as drug abuse, congestive heart failure, cirrhosis, depression/anxiety/psychosis, dementia, diabetes, malignancy or tumor, plegia, and renal disease increased the risk of not being treated. Interestingly, exposure to medications used to treat COPD or HIV (HR = 0.824, p = 0.013) was associated with not being treated (though a diagnosis of HIV increased the risk of being treated). Those with medical contraindications such as acute coronary syndrome, bipolar disorder, hepatic decompensation, retinopathy, hemoglobin < 10 g/dL, and platelets < 50,000 cells/mm^3^ were less likely to be treated.

Correlates of initiation of HCV treatment in U.S. Veterans with evidence of homelessness Nearly 11% of these HCV patients (n = 11,082) were identified as being homeless using administrative criteria described above. Of these, 686 (6.2%) were initiated on treatment during the 5-year study period. Of the 90,362 non-homeless Veterans, 6,653 (7.4%) received treatment for chronic hepatitis C during this period. Overall, homeless HCV patients were significantly less likely to be treated compared to non-homeless patients (6.2% vs. 7.4%, p<0.0001).

Adjusting for observable characteristics in a multivariable model (Table 2), homeless Veterans were 15.1% less likely to receive HCV treatment than those without an indicator for homelessness. In the multivariable regression model for the subset of homeless Veterans (Table 3), a number of measures were significantly associated with receiving treatment. The model indicated that homeless Veterans age 50 or older, those with other or unknown genotype, a diagnosis of drug abuse, exposure to medications used to treat diabetes, and a hemoglobin level of < 10 g/dL were less likely to be treated. HCV genotype 2 or 3 (HR = 1.70, p<0.0001) and other/unknown race (HR = 1.40, p = 0.002) increased the risk of treatment.

**Table 2:** Results of univariate and multivariable Cox Proportional Hazards regression models of initiation of HCV treatment in 101,444 U.S. Veterans with chronic hepatitis C (HCV) diagnosis who were treatment naïve for HCV, 2004–2009.

	Univariate				Multivariable			
			95% CI				95% CI	
Variable	HR	p-value	LL	UL	HR	p-value	LL	UL
**Homeless**	0.753	< .0001	0.697	0.814	0.849	0.000	0.781	0.922
**Demographic variables**								
Age								
< 50 (referent)	-	-	-	-				
50+	0.242	< .0001	0.230	0.255	0.286	< .0001	0.271	0.302
Genotype								
1 or 4 (referent)	-	-	-	-				
2 or 3	1.981	< .0001	1.887	2.080	1.778	< .0001	1.690	1.870
Other/unknown	0.089	< .0001	0.083	0.096	0.089	< .0001	0.082	0.095
Marital status								
Married (referent)	-	-	-	-				
Not married	1.188	< .0001	1.137	1.242	1.195	< .0001	1.141	1.252
Race								
White (referent)	-	-	-	-				
Black	1.712	< .0001	1.621	1.809	1.272	< .0001	1.202	1.347
Other/unknown	1.466	< .0001	1.379	1.558	1.289	< .0001	1.210	1.372
Insurance status								
No insurance (referent)	-	-	-	-				
Medicare	0.790	< .0001	0.738	0.845	1.074	0.043	1.002	1.152
Medicaid	0.912	0.370	0.746	1.115	1.093	0.388	0.894	1.336
Other	1.223	< .0001	1.150	1.301	1.291	< .0001	1.211	1.376
Sex (male)	0.783	< .0001	0.705	0.870	0.783	< .0001	0.705	0.870
**Co-morbid conditions, by ICD-9-CM codes**								
Alcohol	0.790	< .0001	0.752	0.830	0.862	< .0001	0.817	0.909
Chronic fatigue syndrome	1.205	0.564	0.640	2.266	-	-	-	-
Congestive heart failure	0.317	< .0001	0.252	0.399	0.506	< .0001	0.402	0.639
Cirrhosis	0.682	< .0001	0.587	0.793	0.727	0.012	0.567	0.931
COPD	0.762	0.042	0.587	0.990	-	-	-	-
Cardiovascular disease	0.534	< .0001	0.417	0.684	-	-	-	-
Depression/anxiety/psychosis	0.854	< .0001	0.810	0.900	0.918	0.003	0.867	0.971
Dementia	0.305	< .0001	0.169	0.549	0.483	0.019	0.263	0.886
Diabetes	0.697	< .0001	0.657	0.740	0.844	< .0001	0.793	0.898
Drug abuse	0.725	< .0001	0.690	0.763	0.750	< .0001	0.709	0.793
HIV	1.394	< .0001	1.205	1.611	1.314	0.000	1.128	1.530
Liver disease	1.321	0.002	1.109	1.572	1.199	0.042	1.007	1.427
Malig/tumor	0.751	< .0001	0.665	0.848	0.872	0.029	0.771	0.986
OA	0.973	0.363	0.917	1.032	1.063	0.047	1.001	1.128
Plegia	0.653	0.001	0.513	0.831	0.709	0.006	0.555	0.906
Rheumatoid arthritis	0.773	0.060	0.592	1.011	-	-	-	-
Renal disease	0.491	< .0001	0.406	0.593	0.765	0.007	0.631	0.928
Sleep apnea	1.049	0.521	0.907	1.213	1.231	0.006	1.063	1.425
Sleep disorders (other)	1.048	0.370	0.946	1.162	-	-	-	-
Smoking	1.007	0.763	0.963	1.053	-	-	-	-
Ulcer	0.791	0.020	0.650	0.964	-	-	-	-
**Prescriptions for medications to treat the following conditions**								
Alcoholism	0.891	0.469	0.651	1.219				
Cancer	0.871	0.726	0.402	1.886				
COPD	0.665	< .0001	0.606	0.728	0.819	< .0001	0.746	0.898
Dementia	0.376	0.001	0.209	0.677	-	-	-	-
Depression	0.966	0.166	0.920	1.015	1.048	0.079	0.995	1.105
Diabetes	0.707	< .0001	0.664	0.752	-	-	-	-
Drug abuse	0.889	0.373	0.686	1.152	-	-	-	-
HIV	0.800	0.004	0.688	0.930	0.824	0.013	0.708	0.959
Insomnia	1.231	0.007	1.058	1.431	1.206	0.018	1.033	1.409
Sleep somnolence	1.111	0.797	0.497	2.487	-	-	-	-
Smoking	1.220	< .0001	1.137	1.309	-	-	-	-
**Contraindications—comorbidities**								
Acute coronary syndrome	0.446	< .0001	0.315	0.630	0.603	0.004	0.426	0.853
Bipolar disorder	0.739	< .0001	0.636	0.858	0.695	< .0001	0.597	0.808
Hepatic decompensation	0.558	< .0001	0.479	0.651	0.706	< .0001	0.605	0.824
Intractable epilepsy	0.500	0.115	0.212	1.182	0.335	0.070	0.103	1.094
Major depression with suicide attempt	0.916	0.334	0.766	1.095	3.567	< .0001	2.599	4.896
Myocardial infarction	0.252	0.002	0.107	0.596	0.473	0.091	0.199	1.128
Retinopathy	0.671	0.000	0.546	0.825	0.736	0.005	0.596	0.909
								
**Contraindications—laboratory values**								
Hemoglobin < 10 g/dL	0.343	< .0001	0.296	0.397	0.457	< .0001	0.394	0.530
Platelets < 50,000 cells/mm3	0.320	< .0001	0.235	0.436	0.391	< .0001	0.287	0.532

**Table 3:** Results of univariate and multivariable Cox Proportional Hazards regression models of initiation of HCV treatment in 11,082 U.S. Veterans with evidence of homelessness and with chronic hepatitis C (HCV) diagnosis who were treatment naïve for HCV, 2004–2009.

	Univariate				Multivariable			
			95% CI				95% CI	
Variable	HR	p-value	LL	UL	HR	p-value	LL	UL
**Homeless**	-	-	-	-	-	-	-	-
**Demographic variables**								
Age								
< 50 (referent)	-	-	-	-	-	-	-	-
50+	0.231	< .0001	0.196	0.272	0.268	< .0001	0.226	0.318
Genotype								
1 or 4 (referent)	-	-	-	-	-	-	-	-
2 or 3	2.008	< .0001	1.676	2.404	1.699	< .0001	1.407	2.052
Other/unknown	0.075	< .0001	0.056	0.101	0.075	< .0001	0.056	0.101
Marital status								
Married (referent)	-	-	-	-	-	-	-	-
Not married	1.072	0.566	0.846	1.358	-	-	-	-
Race								
White (referent)	-	-	-	-	-	-	-	-
Black	1.522	< .0001	1.267	1.829	1.108	0.294	0.915	1.343
Other/unknown	1.767	< .0001	1.449	2.156	1.398	0.002	1.134	1.725
Insurance status								
No insurance (referent)	-	-	-	-	-	-	-	-
Medicare	0.871	0.391	0.635	1.194	-	-	-	-
Medicaid	0.749	0.362	0.402	1.394	-	-	-	-
Other	0.995	0.980	0.695	1.425	-	-	-	-
Sex (male)	0.848	0.492	0.529	0.014	-	-	-	-
**Co-morbid conditions, by ICD-9-CM codes**								
Alcohol	0.921	0.301	0.789	1.076	-	-	-	-
Congestive heart failure	0.331	0.053	0.108	1.014	-	-	-	-
Cirrhosis	0.864	0.630	0.477	1.566	-	-	-	-
COPD	0.333	0.120	0.083	1.332	-	-	-	-
Cardiovascular disease	0.324	0.112	0.081	1.298	-	-	-	-
Depression/anxiety/psychosis	0.904	0.222	0.768	1.063	-	-	-	-
Dementia	0.384	0.332	0.056	2.654	-	-	-	-
Diabetes	0.660	0.002	0.512	0.853	-	-	-	-
Drug abuse	0.803	0.007	0.684	0.942	0.778	0.002	0.661	0.915
HIV	1.396	0.086	0.954	2.043	-	-	-	-
Liver disease	0.926	0.790	0.525	1.632	-	-	-	-
Malig/tumor	0.553	0.095	0.276	1.109	-	-	-	-
OA	0.959	0.716	0.766	1.201	-	-	-	-
Plegia	0.873	0.813	0.284	2.686	-	-	-	-
Rheumatoid arthritis	0.810	0.764	0.205	3.201	-	-	-	-
Renal disease	0.570	0.208	0.238	1.367	-	-	-	-
Sleep apnea	0.633	0.363	0.237	1.695	-	-	-	-
Sleep disorders (other)	0.879	0.504	0.603	1.283	-	-	-	-
Smoking	0.963	0.627	0.828	1.121	-	-	-	-
Ulcer	0.490	0.156	0.183	1.313	-	-	-	-
**Prescriptions for medications to treat the following conditions**								
Alcoholism	1.043	0.906	0.517	2.106	-	-	-	-
COPD	0.708	0.036	0.513	0.978	-	-	-	-
Dementia	1.021	0.983	0.147	7.091	-	-	-	-
Depression	0.904	0.215	0.771	1.060	-	-	-	-
Diabetes	0.558	< .0001	0.417	0.746	0.684	0.013	0.508	0.922
Drug abuse	1.231	0.351	0.796	1.903	-	-	-	-
HIV	0.665	0.224	0.345	1.283	-	-	-	-
Smoking	1.428	0.000	1.175	1.735	-	-	-	-
**Contraindications—comorbidities**								
Acute coronary syndrome	0.228	0.139	0.032	1.613	-	-	-	-
Bipolar disorder	0.820	0.311	0.558	1.204	-	-	-	-
Hepatic decompensation	0.744	0.255	0.447	1.237	-	-	-	-
Intractable epilepsy	1.147	0.882	0.188	7.003	-	-	-	-
Major depression with suicide attempt	0.635	0.154	0.340	1.185	0.568	0.069	0.309	1.046
Myocardial infarction	0.525	0.512	0.077	3.595	-	-	-	-
Retinopathy	0.266	0.061	0.067	1.061	-	-	-	-
								
**Contraindications–laboratory values**								
Hemoglobin < 10 g/dL	0.254	< .0001	0.132	0.490	0.311	0.001	0.161	0.601
Platelets < 50,000 cells/mm3	0.875	0.725	0.415	1.842	-	-	-	-

## Discussion

In this study of U.S. Veterans diagnosed with HCV and who were treatment naïve during the period 2004 to 2009, we noted an overall treatment initiation rate of 7.4%. As compared to the general U.S. population, this rate appears to be comparable to the estimates of HCV treatment (7–11%) reported by the CDC [28]; though, in general, treatment rates among U.S. Veterans are considered to be higher as reported in a recent review [17]. The rate is significantly higher than those reported in injection drug users in developed countries such as the U.S. and Australia (reviewed in [29]). The rate we report is lower than the 11.8% reported for HCV treatment prescriptions for U.S. Veterans for the period 1999–2003 [14]; possible explanations include the non-overlapping time periods and the evolution of practices related to the diagnosis and management of HCV in the VA.

Our results are consistent with studies that have identified age >50 years, unknown genotype, and low hemoglobin as patient-level factors associated with not receiving HCV treatment in U.S. Veterans [13, 14, 30, 31]. It was interesting that exposure to medications for HIV was associated with not being treated, though a diagnosis of HIV increased the risk of being treated. A diagnosis of major depression with suicide attempt increased the risk of treatment significantly in this cohort, though this is traditionally considered a contraindication.

This is the first study to analyze treatment initiation patterns for a large cohort of homeless HCV patients in the U.S. and does so in the setting of the VA healthcare system, the single largest provider of healthcare services for chronic HCV [19] and homeless patients in the U.S.[32]. The overall rate of initiation of treatment among U.S. Veterans with administrative evidence of homelessness was 6.2%; this is higher than that reported in injection drug users (reviewed in [29]) and comparable to estimates of treatment rates among the U.S. population [28].

Though the rates of initiation of therapy were clinically similar, we found that homeless HCV positive Veterans were statistically less likely to initiate therapy compared to non-homeless HCV positive Veterans. This is most likely because of the high degree of burden that HCV treatment places on the patient. It is likely that clinicians are reluctant to prescribe medications such as interferon and ribavirin that carry a high side effect profile. A number of demographic characteristics, comorbid diagnoses, drug exposures, and contraindications are able to predict initiation of treatment in homeless patients. Our results are similar to the low self-reported rates of ever having received HCV related medical care among homeless adults in Los Angeles [33].

The National Institutes of Health [1], American Association for the Study of Liver Disease [22], and the VA [18] recommend treatment for all eligible HCV patients with moderate or severe disease and that treatment plans can be explored and individualized for patients with mild disease. The initiation of therapy depends on a dialog and understanding between the patient and provider. In the era of shared decision-making, this involves patient factors such as preference, willingness to commit to a harsh regimen of medications and follow-ups, co-morbidities, contraindications, and behavioral factors. In this setting, age may be a proxy for the degree of fibrosis and liver damage. Provider factors such as experience, expertise, and willingness to reach out to the patient also play a key role. Drug abuse may be a correlate for lack of a desire for the practitioner to start treatment. The low rate of initiation of HCV therapy in homeless Veterans is likely due to a combination of these factors. Though geographic and financial barriers have also been cited as significant impediments to initiating HCV therapy [34] for the general population, these theoretically should not have been an issue for Veterans eligible for free care in the VA that includes travel allowance for approved visits [35].

This study provides a profile of the homeless individuals who have been initiated on HCV treatment in the VA healthcare system. Poor adherence and early discontinuation of therapies is common [36–38], with factors such as mental health and substance abuse presenting significant challenges to initiation of therapy. It is important to understand the individual characteristics of patients who receive HCV treatment so that educational and support programs can be designed to increase the risk of adherence to treatment, accounting for factors such as alcoholism, drug abuse, and mental health co-morbidities. Our observation that there were significant differences in age, race, and sex between treated and untreated homeless Veterans with a diagnosis of HCV should be further explored to inform intervention and/or policy strategies for improved treatment uptake.

Additionally, the finding that exposure to medications to treat diabetes predicted lower rates of initiation of treatment is to be noted and further studied as the prevalence of diabetes is high in U.S. Veterans in general. The goals of planned strategies should to be increase the rates of HCV treatment initiation and success in these patients [39, 40].

This study had several limitations. The use of ICD-9-CM codes for assessing co-morbid diagnoses has inherent limitations, though chronic conditions are likely well represented in such administrative data. The collinearity of certain variables such as age and occurrence of chronic conditions such as heart disease and diabetes are implied in our analyses and may pose challenges in interpretation of the results. The design of this study in starting with a cohort of HCV patients and then determining homeless status does not allow us to estimate the overall prevalence of HCV among homeless Veterans. Homelessness was identified using administrative indicators which may not necessarily be complete or reliable. We used a practical time-frame of 6 months prior to the diagnosis of HCV during which time the Veteran was required to have had at least one encounter in the VA and an indicator of homelessness. These criteria may have underestimated the true prevalence of homelessness in this cohort. We were also not able to determine the homeless status of the Veterans during the entire study period. The exact circumstances of homelessness among this cohort was not known (such as street, shelter, transitional housing) nor details regarding whether they were receiving case management or other services. Prescriptions for dual therapy for HCV from pharmacy databases were used as a proxy for treatment initiation. Differences in methodology do not allow us to directly compare our results to those of the “State of Care for Veterans with Chronic Hepatitis C” for homeless Veterans [16]. Further studies are needed to determine true rates of initiation, compliance, and completion of HCV therapy in Veterans experiencing homelessness.

In summary, this is the first study to describe rates and initiation of treatment in a large cohort of U.S. Veterans and demonstrate a relatively lower rate of initiation of therapy in homeless Veterans as compared to housed U.S. Veterans. Apart from well-known contraindications being more prevalent in this cohort, more detailed studies are needed to elucidate specific factors involved, especially as newer therapies have available for HCV. Studies that explore treatment correlations with social determinants of health and intensity of engagement with the healthcare system would likely be important in planning for large scale treatment programs with an emphasis of elucidating similarities and differences between VA and non-VA settings.

Clear benefits associated with these newer therapies such as oral regimens, increased response rate and fewer side effects would likely make it easier to discuss, offer and initiate therapy. However, the issues of patient preference, history of adherence to treatment, and co-morbidities (physical/mental health and laboratory abnormalities) remain. Provider experience with treating HCV, adherence to guidelines [41] and relationship with the patient also remain important factors. Just offering a ‘pill’ may not be enough for sustaining a successful program for treating HCV in homeless Veterans. Furthermore, the newer therapies are several-fold more expensive than IFN-based therapies. The issues of cost-effectiveness and who should be offered these therapies pose new challenges for managing HCV in homeless adults. Lessons learned in the VA will be generalizable to the general homeless population as Veterans are over-represented in this group.
